# Renal Abscess Caused by Crizotinib: A Rare Case Report

**DOI:** 10.3389/fonc.2022.920990

**Published:** 2022-07-07

**Authors:** Zhaojun Wan, Kai Wang, Xiangfu Yin, Xiangting Guo, Guoli Cheng, Jihong Pan

**Affiliations:** ^1^ Department of Oncology, Rizhao Hospital of Traditional Chinese Medicine, Rizhao, China; ^2^ Department of Oncology, The People’s Hospital of Rizhao City, Rizhao, China; ^3^ Department of Pediatrics, Rizhao Hospital of Traditional Chinese Medicine, Rizhao, China; ^4^ Department of Rheumatology and Immunology, The People’s Hospital of Rizhao City, Rizhao, China

**Keywords:** renal abscess, crizotinib, renal cysts, ROS1, NSCLC

## Abstract

Crizotinib is a tyrosine kinase inhibitor that has been found to be effective in the treatment of c-ros oncogene 1-positive non-small cell lung cancer. Although this targeted agent for treating cancer has shown superiority to standard chemotherapy in some ways, this drug has adverse effects, such as the development of renal abscesses. Some associated renal damage may disappear with crizotinib withdrawal. Hence, we present the case of a 58-year-old man with non-small cell lung cancer on crizotinib therapy who developed bilateral renal abnormal space-occupying lesions, successively which were difficult to identify using various imaging methods; even PET-CT highly suspected the right renal masses as malignant. Finally, the right renal lesions were confirmed as renal abscesses by postoperative pathology. The left renal lesion was considered as renal cysts through the lesion disappearing after crizotinib withdrawal. There have been very few reports in this respect, especially proved by various methods and confirmed by postoperative pathology. It is important to recognize this drug-related complication in order to avoid incorrect diagnosis and inadequate therapy. It is necessary to monitor renal changes after taking crizotinib.

## Introduction

Non-small-cell lung cancer (NSCLC) accounts for about 85% of lung cancer cases, and adenocarcinoma was the highest among NSCLCs (1, 2). At present, targeted therapy has become one of the most important treatment methods in NSCLC, especially in lung adenocarcinoma with the mutation of driver genes, including c-ros oncogene 1 (ROS1), whose mutation is a rare molecular driver of NSCLC that can be effectively treated with crizotinib (3). However, crizotinib has been found to have an adverse effect on the development of renal cysts (4, 5), with an incidence of 5% showing in the drug instructions. In Asian patients, the incidence of progression of existing renal cysts and the development of new renal cysts are higher in patients who were treated with crizotinib (4). There were even infected renal cysts during crizotinib therapy (6). In the present case, the right renal lesions were eventually confirmed to be renal abscess formations. Nevertheless, these benign lesions could be easily misdiagnosed as metastasis from lung cancer or a new primary tumor, even with multiple imaging methods. Furthermore, the pathogenesis of this rare adverse event is unclear.

The aim of this study was to describe the presentation and the patient should be closely observed for renal complications during crizotinib therapy, in order to avoid irreversible damage. In addition, we explore the possible mechanisms of crizotinib-associated renal cysts (CARCs). The patient provided written informed consent.

## Case Presentation

**Figure 1:**
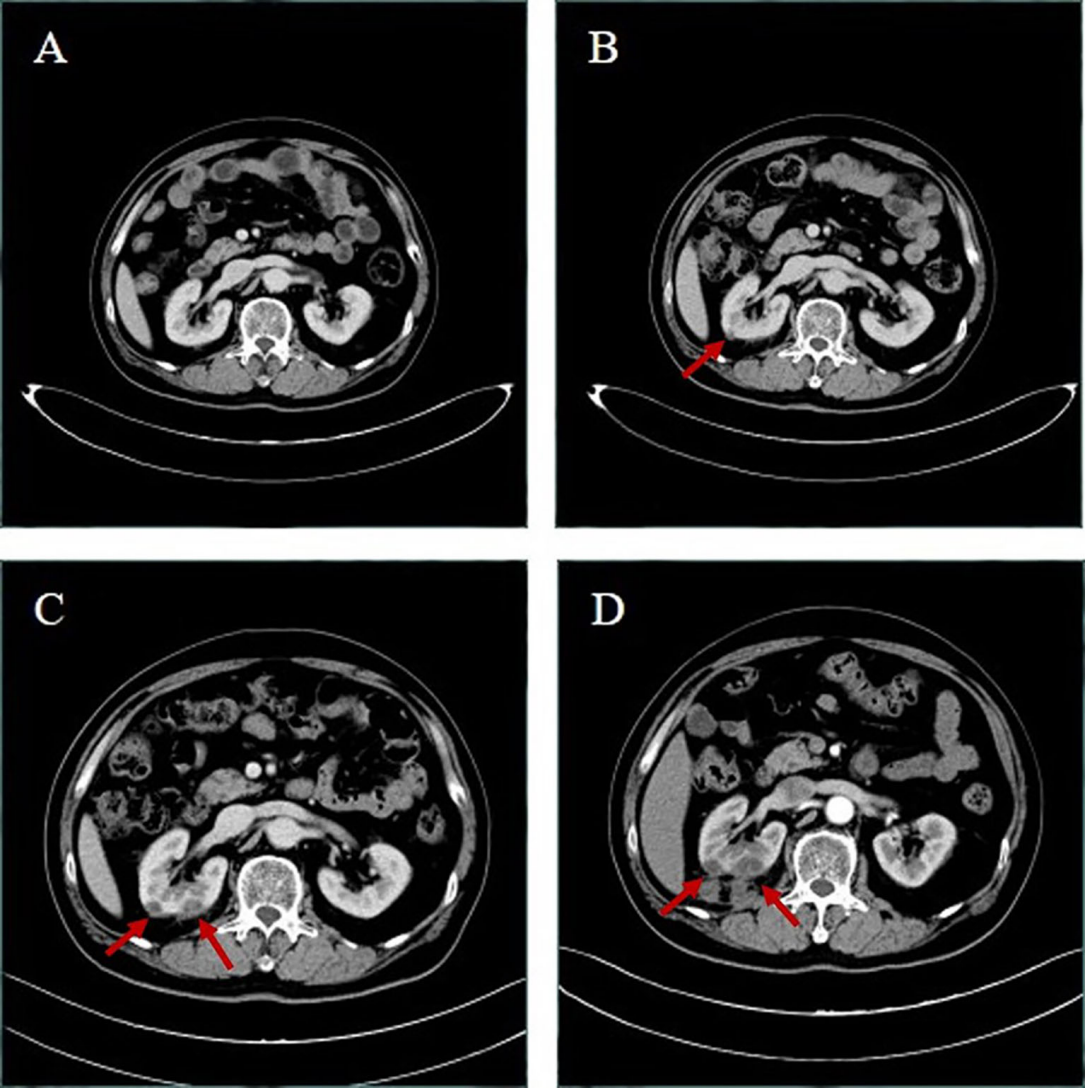
Upper abdominal computed tomography (CT) of the patient. **(A)** After more than 3 months’ therapy with crizotinib in May 1, 2020, both kidneys have uniform density and no abnormity. **(B)** After 5 months’ therapy in June 27, 2020, the right kidney appeared to have new slightly low-density lesions; the larger one was about 10 mm in diameter. **(C)** After more than 6 months’ therapy in August 20, 2020, the right kidney appeared to have slightly low-density lesions larger than in June 27, 2020, and the larger one was about 13 mm in diameter, and the right kidney appeared to have a new low-density lesion, whose diameter was about 18 mm. Upper abdominal enhanced CT in October 2, 2020 **(D)** showed that multiple low-density lesions in and around the right kidney were enlarged and partially new; the larger one was about 24 mm in diameter.

**Figure 2:**
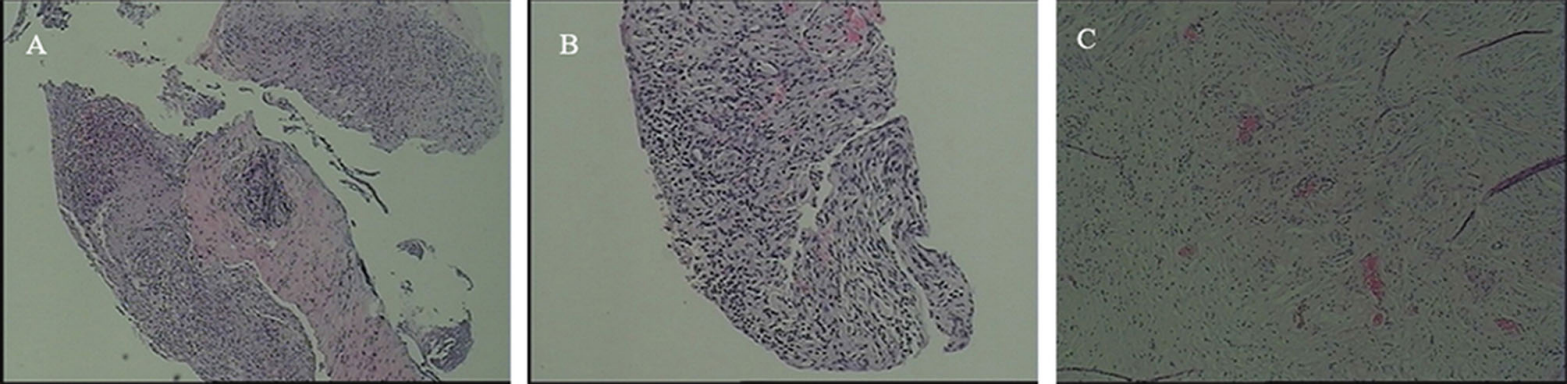
CT-guided biopsy of the right renal lesion in August 24, 2020 and in September 4, 2020, respectively, The pathologic result revealed respectively that **(A)** there was fibrous tissue with extensive inflammatory cell infiltration and **(B)** there was lymphocytic infiltration in the stroma, but no tumor was observed. **(C)** After laparoscopic radical nephrectomy of the right kidney under general anesthesia, postoperative pathological analysis revealed that kidney tissue and perirenal adipose sac presented chronic suppurative inflammation and abscess formation, but still no tumor was observed.

**Figure 3:**
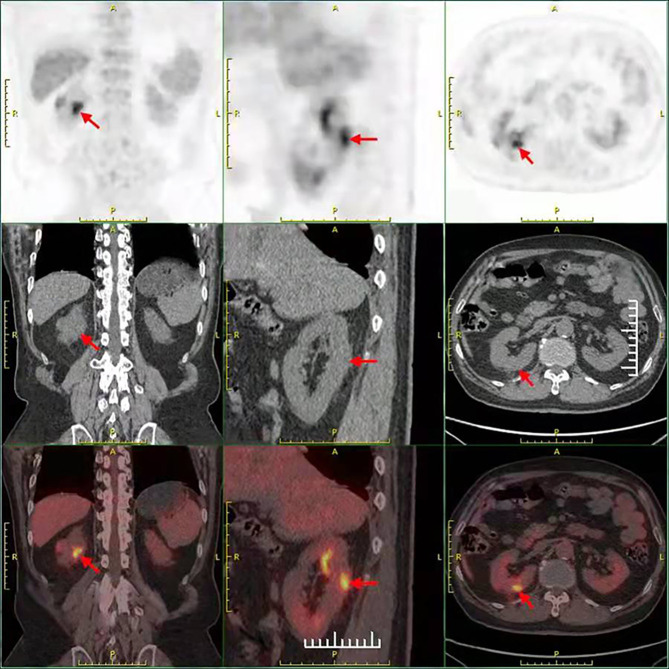
The result of the PET-CT examination in September 1, 2020 showed that there were two low-density foci in the parenchyma of the right kidney. The larger one had a higher metabolism, possibly a malignant lesion.

**Figure 4:**
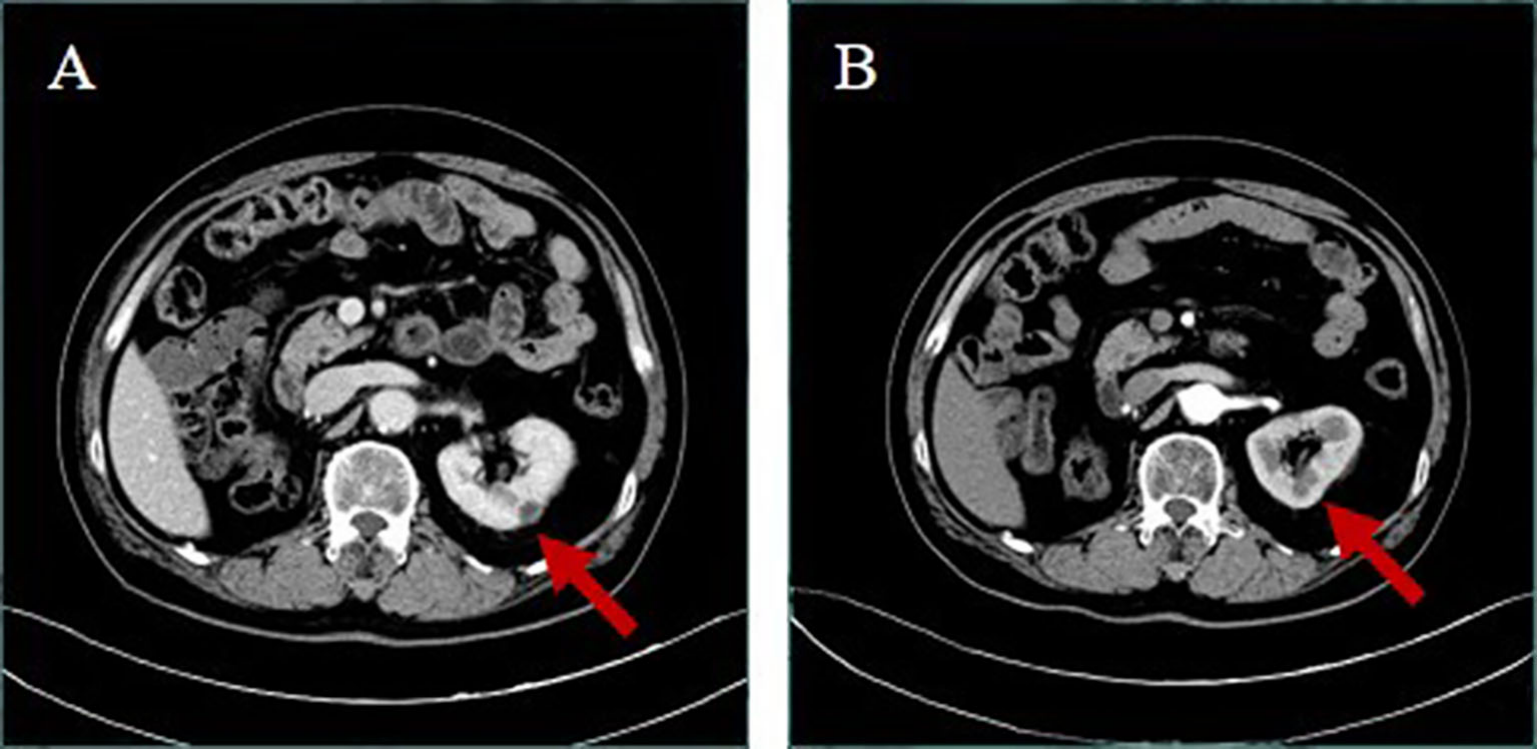
Upper abdominal enhanced CT **(A)** in December 3, 2020; the right kidney was not shown and the left kidney appeared to have a new low-density mass, about 11 mm in diameter, with marginal enhancement. **(B)** In February 18, 2021, after more than 2 months of crizotinib withdrawal, the patient revealed that the abnormal enhancement of the left kidney was not obviously displayed.

The current study presents the case of a 58-year-old man with non-small cell lung cancer, whose pathological type was adenocarcinoma. Through a series of genetic tests, the result shows a ROS1-positive cancer. At the outset, the result of cervical color Doppler ultrasonography reveals multiple lymph node metastasis. Moreover, the patient had Grade 2 hypertension. To begin with, the patient accepted six cycles of pemetrexed combined with carboplatin chemotherapy. The disease was evaluated to have partial remission after two cycles of chemotherapy. During the last four cycles of chemotherapy, the disease was evaluated to be stable. Then, from January 15, 2020, the patient began to accept crizotinib-targeted therapy. Until May 1, 2020, the reexamination of upper abdominal computed tomography (CT) showed that no obvious abnormality was observed in both kidneys (**Figure 1A**). In June 27, 2020, the examination of upper abdominal CT showed that the right kidney was occupied and the other lesions of this patient were evaluated to be stable (**Figure 1B**). In August 20, 2020, the reexamination of upper abdominal CT showed that the right renal lesion (**Figure 1C**), which was suspected to be a malignant tumor, not excluding metastatic tumors, was larger than in June 27, 2020, but the other lesions of this patient were still evaluated to be stable. In order to confirm the diagnosis, the patient accepted needle biopsy of the right kidney in August 24, 2020. The pathologic result did not find tumor cells (**Figure 2A**). For a more cautious diagnosis, the patient accepted PET-CT examination in September 1, 2020, the results of which showed that the mass lesion of the right kidney was likely a malignancy (**Figure 3**). Then, the patient accepted needle biopsy of the right kidney again in September 4, 2020. The pathologic result still did not find tumor cells (**Figure 2B**). In October 2, 2020, the examination of upper abdominal enhanced CT showed that multiple low-density lesions in and around the right kidney were enlarged and partially new (**Figure 1D**) compared to August 20, 2020 (**Figure 1C**). Therefore, the patient was treated with laparoscopic radical nephrectomy of the right kidney under general anesthesia in October 8, 2020. Eventually, postoperative pathological analysis revealed renal abscess formation (**Figure 2C**). The patient continued to take crizotinib orally after the surgery. However, the unfortunate thing happened again. In December 3, 2020, when the patient re-checked enhanced CT of the chest and upper abdomen, although the result revealed that the lung tumor was stable (the right kidney was not shown), the left kidney appeared to have abnormal enhancement (**Figure 4A**). By this time, we realized that the most probable cause of the kidney disease was an adverse reaction to crizotinib. Thus, the patient stopped taking crizotinib. The patient received intensity-modulated radiation therapy for lung cancer beginning in December 21, 2020. After more than 2 months, *via* enhanced CT of the abdomen, the abnormal enhancement of the left kidney was not obviously displayed (**Figure 4B**). Subsequently, the patient discontinued crizotinib treatment, and instead received bevacizumab-targeted therapy combined with pemetrexed chemotherapy. No renal lesions were found during this period. The patient’s case emphasized that we should pay attention to the renal complications of crizotinib.

## Discussion

Crizotinib is a commonly used targeted drug in the treatment of NSCLC with ROS1-positive, anaplastic lymphoma kinase (ALK)-positive, and c-MET-positive results, which have been associated with the development of renal cysts. Crizotinib treatment has been associated with the development of the patient’s complex renal cysts (~4%) (7). Complex renal cysts have a risk of complications such as hemorrhage, rupture, or infection (6). With the development of targeted drugs, crizotinib is increasingly used in patients with related gene mutations in lung adenocarcinoma, but there is little related diagnosis and treatment about crizotinib-associated renal cysts (CARCs).

CARCs progressed from the existing simple renal cysts through most of the reported cases (4, 8, 9), which is different in our study. The current study presents a case of a 58-year-old man with ROS1-positive NSCLC, who was found to have right and left renal lesions successively appearing during treatment with crizotinib. The right renal lesions were postoperatively pathologically confirmed to be asymptomatic renal abscess formations eventually, which could have developed from complex renal cysts caused by crizotinib. The left renal lesion, which was a CARC, disappeared after discontinuing crizotinib treatment for more than 2 months.

Although the present patient developed renal abscess formation on the right kidney as confirmed by postoperative pathology during treatment with crizotinib, the patient did not have fever, backache, and other relevant clinical symptoms, and the imaging studies are insufficient for a diagnosis of renal abscess; even PET-CT showed that the lesion of the right kidney was likely a malignancy. Furthermore, the pathologic result of CT-guided biopsy revealed that there was fibrous tissue with extensive inflammatory cell infiltration the first time and there was lymphocytic infiltration in the stroma the second time, which suggested that the renal abscess of the right kidney was most probably developed by complex renal cysts. All of the above suggests that these renal lesions are difficult to distinguish from renal cysts, renal abscesses, metastases from lung cancer, or new primary tumors. It could be easily misdiagnosed as metastasis from lung cancer or a new primary tumor, even with multiple imaging methods. Most of the CARCs regress spontaneously after crizotinib withdrawal (4, 10). It is consistent with our study in which the left renal lesion, which was a CARC, disappeared after discontinuing crizotinib treatment for more than 2 months. Therefore, enhancing our understanding and the rapid identification of CARCs can avoid unnecessary invasive examinations and treatments. In addition, it can also prevent cysts from developing into abscesses.

The molecular mechanism for the development of CARCs is unknown. Research has shown that renal cysts are thought to be acquired lesions that evolve from diverticula in distal convoluted and collecting renal tubules (11). C-MET receptors, which are the receptors of Hepatocyte growth factor/scatter factor (HGF/SF), are normally present in renal tubular epithelium (12), where it stimulates cell growth (13). A previous study showed that HGF and its receptor c-Met promote cystogenesis (14). HGF-mediated activation of Mapk/Erk and/or Stat3 appears to be an important mediator of renal cystic formation (15, 16). Based on the above analysis, the inhibiting effect of c-MET by the drug action can reduce the incidence of renal cystic formation. However, the fact would be paradoxical. One study showed that inhibition of c-Met by crizotinib found no significant activation of the Mapk/Erk or Stat3 signal pathway in mice treated with crizotinib (17). Accordingly, it could be that an unidentified feedback mechanism increased HGF levels *via* other targets to drive cyst formation when the c-MET was inhibited by crizotinib.

In addition, we found that vessel injury increased FAK activity (18). However, inhibiting FAK interrupted FSK-mediated Src activation and upregulation of ERK and mTOR pathways in an animal model, which is a critical involvement in renal cyst development (19). Based on the above analysis, we think that vessel injury increased FAK activity, which induced FSK-mediated Src activation and upregulation of ERK and mTOR pathways, leading to renal cyst development. Therefore, we suggest that renal cysts are more common in patients with diseases that cause vascular damage after treatment with crizotinib. It is well-known that renal vascular damage is common in hypertensive patients. In this report, the patient has hypertension, controlled by a combination of two drugs. Based on the above analysis, we realize that the incidence of renal cyst as a complication in patients with hypertension taking crizotinib may be different from that in patients without hypertension. Furthermore, one study showed that angiotensin-converting enzyme 2 inhibits FAK expression (20). FAK has been described above to have an important role in the development of renal cysts; thus, we suspected that the incidence of renal cyst as a complication in patients with hypertension being treated with angiotensin-converting enzyme inhibitors and who are taking crizotinib is higher than that in patients without angiotensin-converting enzyme inhibitor treatment.

To the best of our knowledge, the present case is the first reported example of a renal abscess formation confirmed by postoperative pathology during crizotinib treatment. Through this report, we need to recognize the CARCs in order to avoid incorrect diagnosis and inadequate therapy. Although we do not fully understand the reason associated with the development of renal cysts after taking crizotinib for NSCLC, it is worthy of further verification in the future. We will continue to explore related problems in future studies.

## Data Availability Statement

The original contributions presented in the study are included in the article/supplementary material. Further inquiries can be directed to the corresponding author.

## Ethics Statement

Written informed consent was obtained from the individual(s) for the publication of any potentially identifiable images or data included in this article.

## Author Contributions

ZW wrote the manuscript. KW and XG were involved in diagnosing, treating, providing follow-up for the patient, and collecting data for this report. XY revised the manuscript. GC and JP participated in the analysis of the case. All authors read and approved the final version of the manuscript for submission.

## Conflict of Interest

The authors declare that the research was conducted in the absence of any commercial or financial relationships that could be construed as a potential conflict of interest.

## Publisher’s Note

All claims expressed in this article are solely those of the authors and do not necessarily represent those of their affiliated organizations, or those of the publisher, the editors and the reviewers. Any product that may be evaluated in this article, or claim that may be made by its manufacturer, is not guaranteed or endorsed by the publisher.
